# Incisional hernia prediction using machine learning models

**DOI:** 10.1186/s12911-026-03382-8

**Published:** 2026-02-27

**Authors:** Edgard Efren Lozada-Hernández, Tania A. Ramirez-DelReal, Sebastián Salazar-Colores, Dagoberto Armenta-Medina

**Affiliations:** 1 Centro de Investigación e Innovación en Tecnologías de la Información y Comunicación (INFOTEC), Aguascalientes, 20326 México; 2 Servicios de salud del Instituto Mexicano del Seguro Social para el Bienestar (IMSS-BIENESTAR) Hospital Regional de Alta Especialidad del Bajío, León, Guanajuato, 37660 México; 3 Secretaría de Ciencia, Humanidades, Tecnología e Innovación (SECIHTI), Investigador por México, Ciudad de México, 03940 México; 4 Centro de Investigación en Ciencias de Información Geoespacial, Aguascalientes, 20313 México; 5 Centro de investigaciones en Óptica A.C. (CIO), León, Guanajuato, 37150 México

**Keywords:** Midline Laparotomy, Incisional hernia, Machine learning, XGBoost, Regression logistic, Decision tree

## Abstract

**Background:**

One of the main complications after laparotomy is incisional hernia (IH), with an incidence of 40% in specific risk groups. There is no consensus on determining which patients are at low or high Risk. There is no currently full peer-reviewed study in which Machine learning (ML) is applied for the prediction of IH, so the objective of this study was to develop a predictive model of IH based on different ML techniques.

**Methods:**

Retrospective cohort study type of diagnostic tests. Patients over 18 years of age who underwent midline laparotomy were included. The main outcome was the occurrence of IH. Three ML methods were evaluated: Logistic Regression, Decision Tree, and XGBoost. Each model’s predictive capacity, Friedman range score, and clinical utility were assessed. The usefulness of all models was further evaluated using Bayes’ theorem to determine the change in prevalence after applying the models.

**Results:**

789 patients were analyzed, 161 (20.1%) presented IH, the model was built with 13 predictor variables, identifying preoperative risk of surgical site infection as the strongest risk factor. For the XGBoost model, the best performance with an AUC of 0.93 ± 0.02 and a Brier score of 0.10 was achieved. Its clinical utility, confirmed by Bayes’ theorem, showed a positive test raises the posterior probability to 86% (above treatment threshold), while a negative test reduces it to 2%.

**Conclusions:**

A predictive model was created using the XGBoost technique. The robustness of the model is related to the correct selection of the variables. Cross-validation and a learning curve indicated that the model performs robustly and shows potential for generalization to new data. An easy-to-use web application was developed that does not require sophisticated or expensive clinical studies.

**Trial registration:**

The study was registered with the hospital research and research ethics committees of the Regional Hospital of High Specialty of Bajio IMSS Bienestar under registration number CEI/HRAEB/002/2021. It was also registered on the Clinical Trials platform with registration number NCT 05718999 on February 27^th^, 2023.

**Supplementary Information:**

The online version contains supplementary material available at 10.1186/s12911-026-03382-8.

## Background

One of the main complications after laparotomy is incisional hernia (IH), with an incidence of 10–23% [1], and until 40% in specific risk groups [2]. IH is often asymptomatic; however, it can significantly cause morbidity (pain) and harm the patient’s quality of life and body image [3]. The European Hernia Society (EHS) dictates the current recommendations for abdominal wall closure [4, 5]; the interest of these guidelines is to reduce this incidence. The prevention of IH focuses on three strategies: 1. Prehabilitation, 2. Mesh prophylactically [2, 3, 6–12] and 3. Modifying the closure technique in laparotomy [1, 13–15].

However, in IH prophylaxis studies, there is no consensus on determining which patients are at low or high risk, and the determination of this risk is highly variable, making it difficult to compare results, which in turn has led to a lack of consensus on the technique of choice for the prevention of this complication [16]. In the last ten years, an effort has been made to unify criteria, and eight IH predictor scales [17–24] have been published. These assess perioperative risk factors; however, their use has not been generalized because some studies have a small sample size or because the cohorts where they are evaluated are from past decades and do not reflect the current risk of the patient since, in recent years, there have been changes in the technique, the approach (laparoscopic and open) and the materials that are used for abdominal wall closure and have positively impacted the reduction of this complication. These scales have used traditional methods such as multiple logistic regression or Cox regression, which, although validated and tested methods, have at least resulted in the prediction of this complication with low prognostic performance [25].

Artificial intelligence (AI) and machine learning (ML) have become essential tools in the field of medicine, aiding in the diagnosis and treatment of various disorders. [26]. Recent improvements in automated learning systems have demonstrated greater prediction skills in comparison to conventional models. The growth in statistical theory has led to these advancements, since it allows for the identification of non-linear correlations between predictors and the response variable, as well as the management of missing data. Efficiently and flexibly merge underperforming prediction models to create a more accurate one [27, 28] XGBoost, or Extreme Gradient Boosting, is a widely used technique in the domain of supervised machine learning [29–31].

Predicting the patient who will develop a hernia is complicated by traditional statistical techniques. Nevertheless, specialists have demonstrated efforts to introduce artificial intelligence into the sector to improve the quality of care and predict complications before they occur to prevent adverse consequences. So far, 21 articles related to hernia surgery have been published, of which seven (33.3%) focused on AI in inguinal hernia surgery, six (28.5%) AI in abdominal hernia surgery, five (23.8%) on AI in incisional hernia surgery, two (9.5%) on AI in medical imaging and robotics in hernia surgery, and one (4.7%) in the prediction of abdominal wall dehiscence [25, 31, 32].

Although preliminary efforts have explored machine learning for IH risk predictions such as a conference abstract using automated machine learning in a mixed cohort of open and laparoscopic abdominal surgeries [33] and a subsequent study focusing on CT image-based biomarkers [34] no full peer-reviewed study has applied advanced machine learning techniques specifically to predict IH in patients undergoing midline laparotomy. Thus, the objective of this study was to develop a predictive model of IH based on ML techniques (including XGBoost) using clinical variables in this specific population and, secondarily, to develop a web application that presents the model’s results to facilitate clinical use.

## Methods

### Type of study

The results of this study were conducted and reported using the TRIPOD statement (Transparent reporting of a multivariable prediction model for individual prognosis or diagnosis) guide [35]. It was registered with the hospital research and research ethics committees of the Regional Hospital of High Specialty of Bajio IMSS Bienestar. The registration number was CEI/HRAEB/002/2021, also on the Clinical Trials platform, with registration number NCT 0571899. Retrospective, observational, longitudinal cohort study, type studies on prognosis.

**Patients:** Inclusion criteria: General surgery patients over 18 years of age, postoperative midline exploratory laparotomy, who underwent urgent or scheduled surgery, regardless of their background diagnosis, between January 2010 and December 2016, and who completed 24 months of follow-up were included after the initial surgery. Exclusion criteria: patients who did not have complete data in the electronic file, patients who did not complete the follow-up or who did not have imaging studies to corroborate the diagnosis of incisional hernia and patients managed with an open abdomen at the end of the surgical procedure

### Data collection

Variable selection was informed by a meta-analysis of eight established incisional hernia risk scales, yielding 245 candidate variables. These were reduced in three phases: (1) exclusion of variables not meeting inclusion criteria (179 remaining); (2) removal of redundant or synonymous variables (34 global variables); and (3) selection of variables with significant associations in ≥ 2 scales, resulting in 13 final predictors (Appendix A.1).

Wound infection is one of the predictor variables that in the literature has shown a significant interaction with the presence of incisional hernia. Our study is retrospective, and we have accurate information on which patients presented or did not present this complication. However, prospectively, we do not know which patients will have or will not have this variable. Therefore, it was decided to include a risk model for its occurrence in the overall model. This way, when a surgeon uses the model, they can integrate this risk with the general risk of presenting an IH. The Risk wound infection was defined according to the score obtained using the American Association of Surgeons’ web application. The ACS NSQIP Surgical Risk Calculator estimates the chance of an unfavorable outcome (such as a complication or death, including the surgical site infection) after surgery. The risk is estimated based on information the patient gives the healthcare provider about their prior health history. The estimates are calculated using data from many patients who had a surgical procedure similar to the one the patient may have [36]. Appendix A.2 shows the diagnostic performance of the ACS scale.

### Data preprocessing

Following these selection criteria, a subset of variables was chosen to be integrated into the hernia prediction model. The input data for the training of the models were structured and tabulated between 13 predictor variables and one outcome: Variable type: Dichotomous: Sex (Male/Female), COPD, Jaundice, Emergency surgery, Colonic surgery, Anemia, Cancer, Ascites, Previous abdominal surgery, Stoma creation (yes/no). Quantitative: Age (years), Risk of infection (score in the ACS calculator) and BMI (body mass index) (kg/m^2^). The data were obtained from the electronic record, clinical history, anamnestic data, physical examination, and imaging details were collected to determine the presence of IH. Data from the hospital clinical record were obtained from: the klinik system, based on the international system of ICD-10 diseases. With the following keys: ICD 10. K43: Ventral hernia (43.1, 43.2, and 43.3) and ICD 9. 54.11 Exploratory laparotomies. Cases with insufficient data were excluded from the analysis.

Missing data were handled by excluding patients with incomplete records for any predictor variable or outcome, ensuring a complete-case dataset for model training and evaluation.

### Statistical analysis

They were grouped into two classes: with and without hernias. Descriptive statistics were performed: qualitative variables were reported as frequency and percentage, and comparisons between groups were performed with a χ^2^ test. Quantitative variables were subjected to the Kolmogorov-Smirnov normality test with Lilliefors correction.

Normally distributed quantitative variables were reported as mean ± SD and compared using Student’s t-test. Non-normal variables were reported as median (25th–75th percentile) and compared using the Mann-Whitney U test. All comparisons were further adjusted by bivariate logistic regression.

After constructing and evaluating the model, we implemented a web application to facilitate its use in a clinical setting. This application was developed using Django, a high-level Python framework that promotes fast, clean, and pragmatic development [37, 38], and Bootstrap, an open-source toolkit for development with HTML, CSS, and JS [39, 40].

### Sample size selection

The response variable on which the models were trained was binary, where the positive class was the presence of IH, and the negative class was its absence. According to the EHS guidelines, IH was defined as a mass in the abdominal wall with or without ejection of viscera or palpable at the surgical site determined by clinical examination [4]. All patients underwent computed tomography in case of diagnostic doubt, which currently represents the gold standard for diagnosing this complication.

The sample size was calculated using the pmsampsize R package [41] for developing a binary outcome prediction model (not for estimating incidence). The calculation was based on the final number of predictors (13 variables) and the following parameters: target c-statistic (AUC) of 0.90, expected prevalence of incisional hernia of 0.21, Cox-Snell R^2^ of 0.33, shrinkage factor of 0.05, and intercept margin of error of 0.05.

This yielded a minimum required sample size of 349 patients (with at least 74 events), corresponding to an events-per-variable ratio of approximately 5.23 (Appendix A.3). The final dataset included 789 patients (161 events), well exceeding this threshold. The variable reduction process (from 245 to 34 and then to 13 predictors) was performed after the sample size calculation and did not affect the adequacy of the model.

### Model training

During data preparation, the starting algorithm developed two conditions: missing data and a skewed class distribution. We got rid of the cases where there was missing data, and we looked at the different models by balancing the data with the SMOTE technique (synthetic minority oversampling technique) [42] in the training set to oversample the cases with IH. However, the model was built with the original base because balancing did not make the model better at predicting the future. We developed the predictive models using three machine learning algorithms: decision trees, logistic regression, and XGBoost. We processed all the data by writing custom code in Python within the Jupyter Notebook environment (anaconda 3), using the Scikit-Learn [43], XGBoost [44], NumPy [45], Pandas [46], and Matplotlib [47] libraries from Python 3 [48].

In the three algorithms the total population was divided into 75% training and 25% testing. Three models were built with 5-fold cross-validation, and the best model was determined with the Friedman ranking test [49].

The regression model was done with the use of the parameters: C:10, max_iter:100, penalty: l2 and, solver: lbfgs, the validation of the model and the search for the best hyperparameters was done using the GridSearchCV library. The hyperparameters obtained as the best were: C:(0.001, 0.01, 0.1, 1, 10, 100), solver: (‘newton-cg’, ‘lbfgs’, ‘liblinear’), penalty: (l2) and, max_iter: (100, 200, 300).

In the decision tree model the following parameters were used: criterion: gini, entropy, max_depth: None, min_samples_split: 2, min_samples_leaf: 1. The model was validated by searching for the best hyperparameters with the GridSearchCV library, the resulting hyperparameters were: criterion: (gini), (entropy), max_depth: range (1, 15), min_samples_split: range (2, 10) and, min_samples_leaf: range (1, 5) [48].

XGBoost was built with the following parameters: n_estimators = 200, n thread = 2, seed = 444, scale_pos_weight = 1, objective = binary:logistic, and the following hyperparameters obtained with the use of the library GridSearchCV: max_depth: (3, 4, 5), learning_rate: (0.1, 0.05, 0.01), gamma: (0, 0.1), min_child_weight: (1, 5, 10), colsample_bytree: (0.5, 0.8) and, subsample: (0.5, 0.8). And the loss function was the same as that used in the logistic regression [29, 50].

**Model evaluation** After training in the training set, all the models were evaluated in the testing set. Model performance was compared using metrics of discrimination and calibration. Discrimination was assessed by the area under the receiver operating characteristic curve (AUC). Additionally, the Accuracy, Recall, Precision (PPV), Negative Predictive Value (NPV), F1 Score and Specificity of models were also assessed. The best-performing machine learning model was decided based on the combination of the highest AUC and the Friedman Range [51].

**Clinical Use** To evaluate the clinical usefulness of the models the Bayesian analysis was incorporated, measuring the post-hoc prevalence when the test was positive and when it was negative. This calculation is based on determining the positive and negative likelihood ratio, based on the sensitivity and specificity results obtained from the general confusion matrix of each model. Bayes’ theorem measures the posteriori prevalence with the following formula:

$$\begin{gathered} {\mathrm{Posteriori}}\,{\mathrm{prevalence}}\, \hfill \\ {\text{ = }}\,[ {{\mathrm{PLR*P}}( {{\mathrm{Hernia}}} )} )\,{\mathrm{/}}\,( {\mathrm{PLR*P}}\,( {{\mathrm{Hernia}}} )\,\hfill \\ {\text{ + }}\,{\mathrm{NLR*}}( {{\text{1 - P}}\,( {{\mathrm{Hernia}}} )} ) ] \hfill \\ \end{gathered}$$

Where: PLR positive likelihood ratio, NLR negative likelihood ratio. P(Hernia) is the a priori prevalence of hernia obtained from the data set = 0.21. This gives you certainty about the reality and implementation of the results [52].

**Model Explanation** To explain the models, importance graphs were made of the characteristics or variables that participate with a higher percentage in the prediction of the target variable. Each model calculates this graph in a particular way. Logistic regression determines the importance of the characteristics from the model coefficients, which reflect both the magnitude and direction of each prediction. In the decision trees, this importance is calculated as the total reduction in impurity contributed by that variable in each node; this measure is averaged. XGBoost feature importance is measured in the total count at which this feature is used to split the trees and the number of observations that relate to this feature. For the models, these values were calculated, and bar graphs were generated to compare them in the same way and easily interpret the variables that make up each model [53].

## Results

### Patient characteristics

**Fig. 1:**
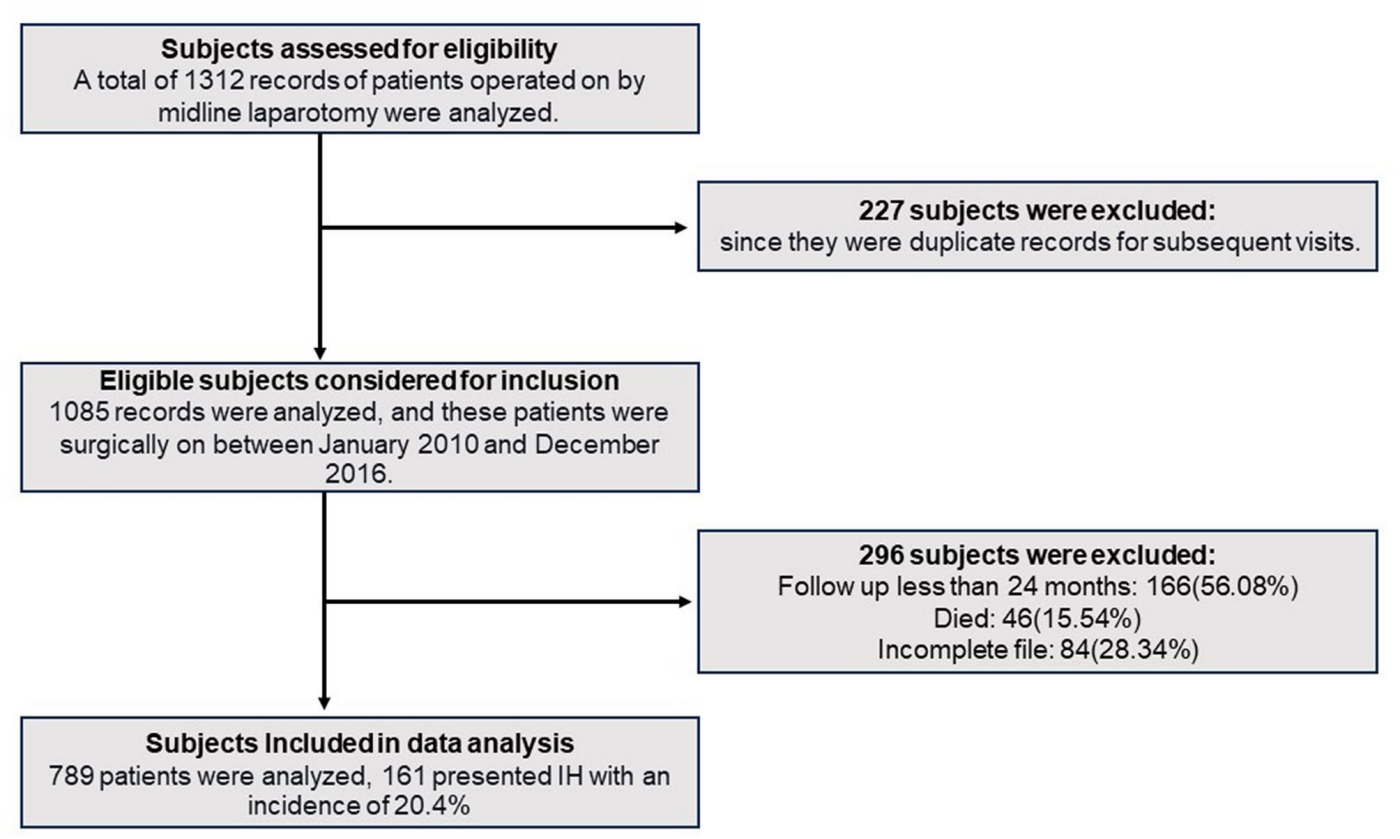
Flow diagram of included subjects

A total of 1312 records of patients operated on by midline laparotomy were analyzed; after removing 227 duplicate records for subsequent visits of the same patient, of the remaining patients, two hundred ninety-six patients were excluded because: they died, the file was incomplete, or they did not complete the follow-up for various reasons, which according to the EHS guidelines, was established for 24 months. Of the 789 patients analyzed, 161 presented IH with an incidence of 20.4%. Figure 1 shows the flow diagram of the included study subjects.

The sample was divided into two groups according to whether or not they showed IH. The association of the predictor variables with the presence of IH was analyzed, a bivariate analysis was performed and adjusted to the bivariate logistic regression, and the goodness of fit was analyzed; Table 1 presents the data.

**Table 1:** Comparison of predictor variables of incisional hernia

Variable	IH n = 161	Without IH n = 628	P	OR	IC al 95%
Age	52.37(14.41)	48.94(16.22)	0.009 *	1.01	1–1.02
Sex					
Female Male	98(60.9) 63(39.1)	428(68.2) 200(31.8)	0.07	0.87	0.56–1.34
Colon Surgery	56(34.8)	141(22.4)	0.001	1.29	0.73–2.19
COPD	4(2.5)	11(1.8)	0.54	1.62	0.42–6.15
Ascites	18(11.2)	37(5.9)	0.400	0.85	0.45–1.58
Jaundice	12(7.5)	37(5.9)	0.464	1.22	0.55–2.72
Anemia	20(12.4)	47(7.5)	0.045	1.64	0.85–3.17
Emergency Surgery	55(34.2)	118(18.8)	0.0001	1.82	1.48–2.89
Wound infection	55(34.2)	56(8.9)	0.0001	2.72	1.63–4.56
Risk wound infection	11.3(9–13.5)	3.2(2.5–4.1)	0.001**	3.5	3.06–3.98
Previous Surgery	126(78.2)	293(46.6)	0.00001	2.65	1.67–4.19
BMI	27.5(5.8)	25.79(4.8)	0.00001*	1.08	1.04–1.12
Ostomy	56(34.78)	124(19.7)	0.00001	1.13	0.65–1.98
Oncologic disease	110(68.3)	472(75.1)	0.07	0.74	0.44–1.25

Bivariate analysis and logistic regression adjusted. *Mean (SD) comparison with Student’s t-test for independent groups. ** Median (percentiles 25–75%) comparison U Mann Whitney. Qualitative variables reported as frequency (percentage) comparison between groups was performed with the χ2 test. COPD: Chronic Obstructive Pulmonary Disease

### Performance of machine learning models

**Fig. 2:**
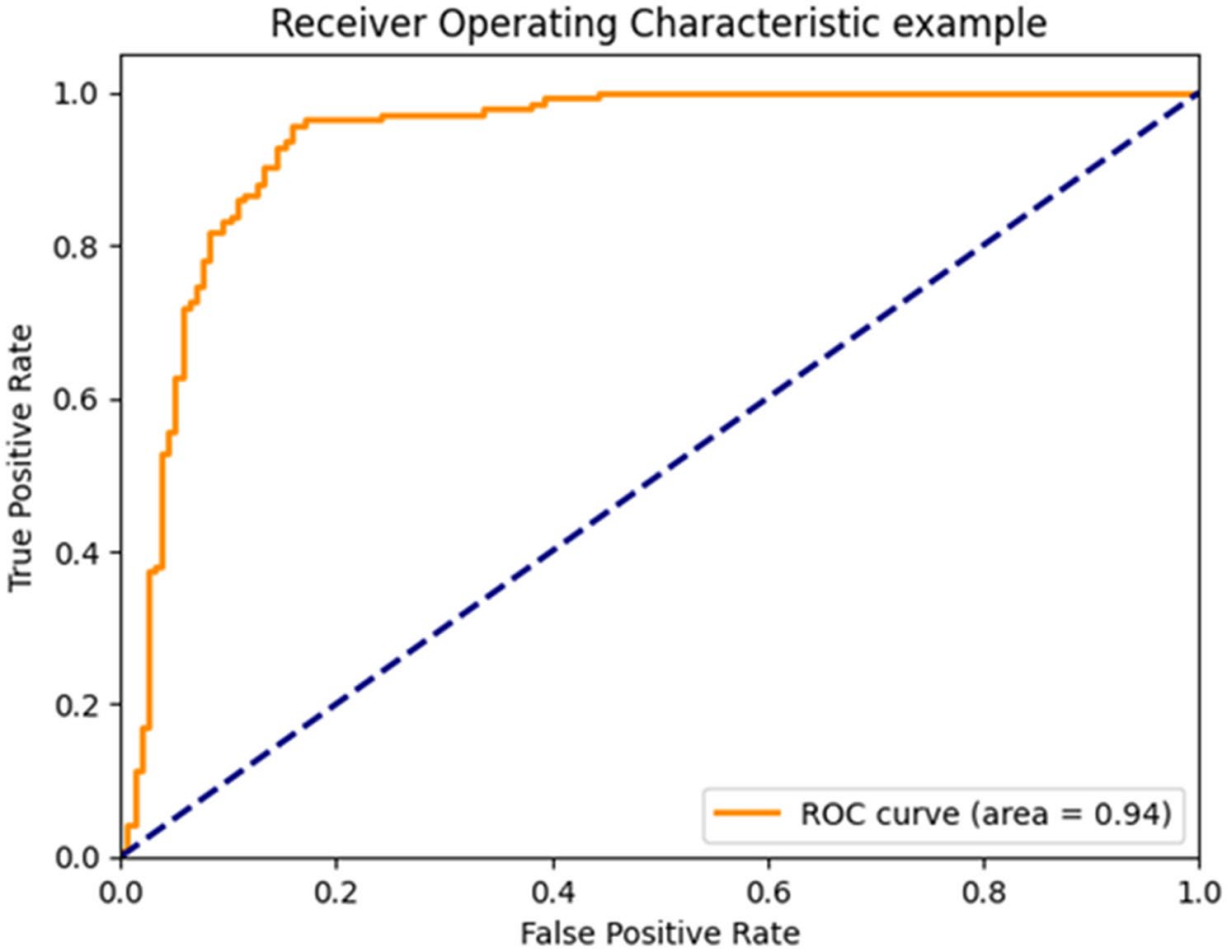
AUC XGBoost model

**Model comparison.** Three machine learning classification techniques were evaluated, and Table 2 presents the results obtained. In general, the Decision Tree model presented lower metrics compared to the other two models; however, these differences were minimal and without reaching a statistically significant difference between the models, so to objectively and statistically robustly evaluate the differences in performance between the techniques analyzed, the Friedman test was applied, a non-parametric statistical technique suitable for comparing multiple groups on paired samples. This method was used because of its effectiveness in handling metric variations between models without assuming a normal distribution of the data. The result was a score that was higher for the XGBoost 2.5 technique, the Friedman statistic resulted in: 5.70 *p*-value: 0.0578. The accuracy for the models were: XGBoost 0.95, Decision Tree 0.94 and Logistic regression 0.95, but the recall were: 0.90, 0.87, 0.87, respectively, from the medical point of view in terms of screening, recall turns out to be the most useful measure. So, we decided together with Friedman’s ranking that the XGboost model would be chosen as the final model. Figure 2 show the ROC curve for XGboost model.

**Table 2:** Comparison of models

Metric	XGBoost	Decision tree	Logistic regression
**ROC AUC**	0.93(0.02)	0.91(0.03)	0.92 (0.3)
**Accuracy**	0.95(0.01)	0.94(0.02)	0.95(0.01)
**Recall**	0.90 (0.05)	0.87(0.08)	0.87(0.06)
**PPV**	0.88(0.03)	0.84(0.08)	0.92(0.05)
**NPV**	0.97(0.01)	0.97(0.02)	0.92(0.05)
**F1 score**	0.89(0.02)	0.86(0.05)	0.89(0.04)
**Specificity**	0.97(0.01)	0.97(0.03)	0.97(0.01)
**AUPRC**	0.81(0.04)	0.80(0.04)	0.83(0.06)
**Friedman Ranking**	2.5	1.42	2.07

ROC AUC: Area Under the Receiver Operating Characteristic Curve, AUPRC = Area Under the Precision-Recall Curve, PPV: Positive predicted value, NPV: Negative predicted value. Reported: mean (Standard deviation)

In summary, XGBoost outperformed logistic regression and decision tree models across key metrics (Table 2).

### XGBoost model interpretation and ablation analysis

**Fig. 3:**
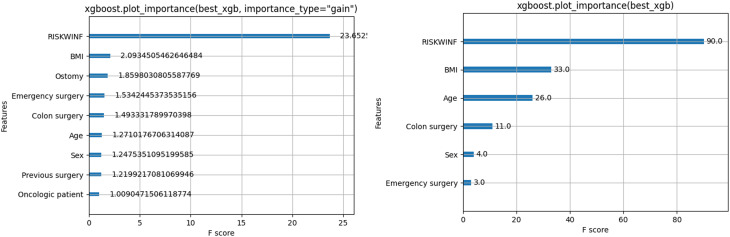
Features important gain and cover

The importance of each variable in the model was measured by its contribution to the prediction. In Fig. 3, two types of importance measures are shown: F score and cover. RISKWNF: This variable has the highest F score, indicating it is the most critical feature in the model. It significantly influences the prediction. BMI, Previous Surgery and Age also have high F scores, showing they play important roles in the model.

In these results we found that the variable risk of infection was the most important variable in the model, even up to five times greater than the next variable BMI. Therefore, it was decided to do an ablation analysis, with the purpose of understanding the contribution of each component and how the absence of certain characteristics affects the precision, overfitting, or generalization capacity of the model. With the goal of removing one or more features without changing its accuracy, and optimizing it to simplify it, possibly improve execution times and reduce the complexity of the model to improve generalization to new data.

**Fig. 4:**
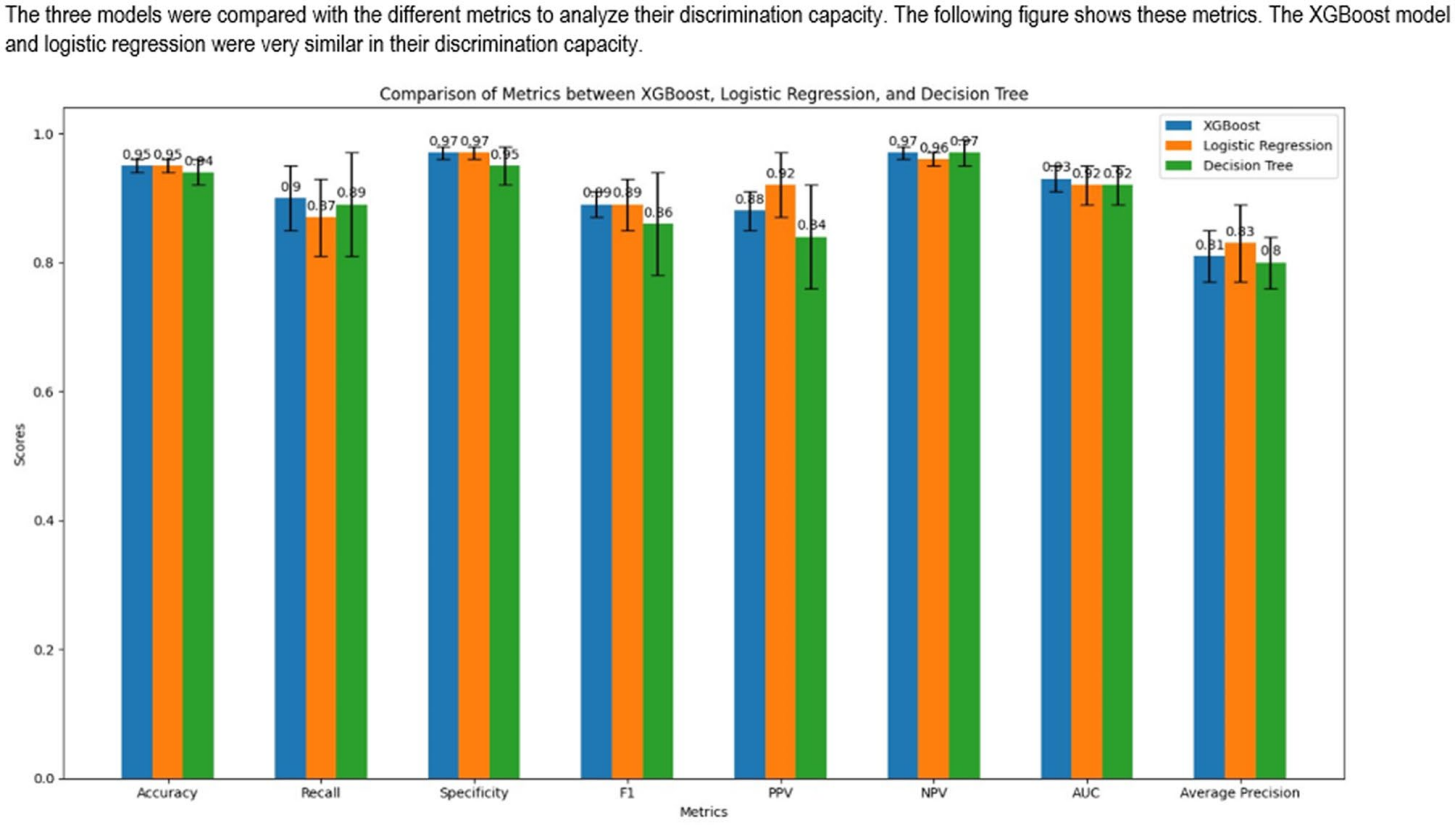
Ablation study

In this analysis, three other models were chosen, 1. only the infection risk variable, 2 without the infection risk variable and 3. with the most important variables from the importance and gain analysis carried out previously (Infection risk, BMI, Age, Emergency surgery and previous surgery). The results of this analysis are shown in Table 3 and Fig. 4.

**Table 3:** Ablation analysis

Metric	Original	Infection	No Infection	Important Variables
ROC AUC score	0.93 (0.02)	0.92 (0.02)	0.55 (0.02)	0.93 (0.03)
Accuracy score	0.95 (0.01)	0.94 (0.01)	0.76 (0.05)	0.95 (0.01)
Recall score	0.90 (0.05)	0.88 (0.05)	0.20 (0.04)	0.90 (0.05)
PPV score	0.88 (0.03)	0.86 (0.03)	0.43 (0.13)	0.88 (0.2)
NPV score	0.97 (0.01)	0.97 (0.01)	0.81 (0.1)	0.97 (0.01)
F1 score	0.89 (0.02)	0.87 (0.03)	0.26(0.04)	0.88 (0.02)
Specificity score	0.97 (0.02)	0.96 (0.01)	0.90 (0.07)	0.96(0.01)
Friedman Score	3.57	2.2	1	3.2

The Original, Infection and Important Variables models perform similarly and fairly well across all metrics. The model No infection, however, shows a significant decrease in almost all metrics, especially Recall, F1-Score and AUC, indicating much poorer overall performance.

Friedman Test Results Average Ranks of each Model: Complete: 3.57, Infection Only: 2.21, No Infection: 1.00, Important Variables: 3.21 The average ranks reflect the relative performance of each model, with a higher rank indicating better performance. In this analysis, the “Original” and “Important Variables” models show the highest ranks, suggesting that these perform better compared to the “Infection” and “No Infection” models.

Friedman statistic and *p*-value: Friedman statistic: 19.23 *p*-value: 0.000245. A significantly low *p*-value (less than the standard threshold of 0.05) indicates that there are statistically significant differences between the evaluated models. This implies that not all models present the same level of performance according to the evaluated metrics. The Nemenyi-Friedman post-hoc test was performed to identify which models this difference is between: Complete models and important variables show high *p*-values (*p* = 0.9), which suggests that there are no differences between these models. The Infection Only (*p* = 0.01) and No Infection (*p* = 0.05) Models show statistically significant differences when compared to the others.

We decided to opt for the complete model since it presented the best metrics and, in the Friedman, score it had the best average range. Although in the post-hoc analysis, the original model does not present differences with the model of important variables, the latter discards clinically significant variables such as ostomy, colon surgery, anemia, ascites, and jaundice that contribute to the model in a very similar way to the others, as shown. Observed in the importance plot since its exclusion in the model without infection results in a notable decrease in predictive capacity.

**Fig. 5:**
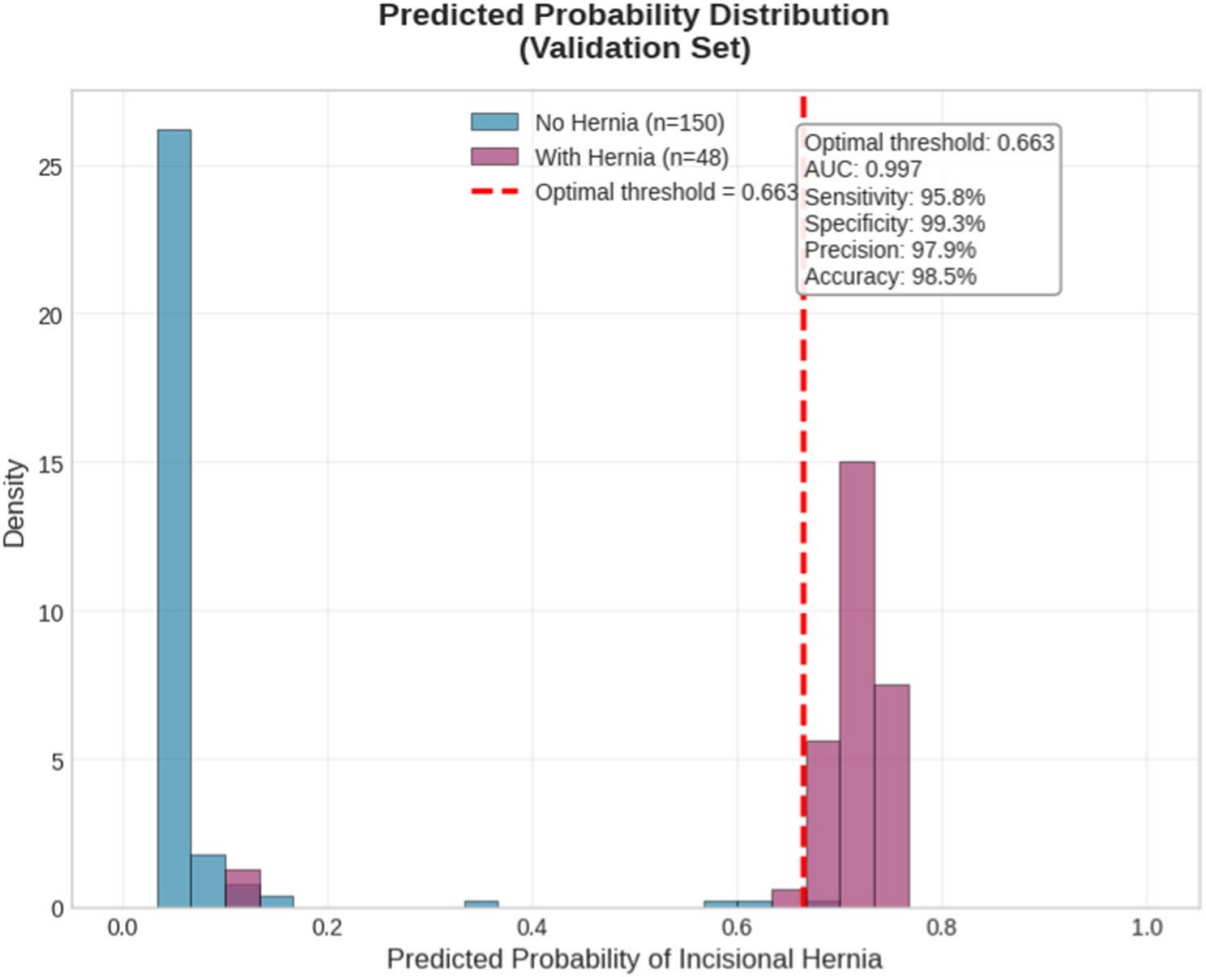
Optima threshold

Based on Youden’s index analysis, the optimal threshold for classifying patients as high risk for incisional hernia was 0.663. At this point, the model demonstrated balanced performance with 95.8% sensitivity and 99.3% specificity. Patients with predicted probabilities ≥0.663 should be classified as high risk for developing incisional hernia. Figure 5 shows the Optimal Threshold.

### Bayesian analysis, calibration and decision curve

In medicine, Bayes’ theorem is a tool used to calculate the probability of an event occurring given prior knowledge of it. Bayes’ theorem calculates the a posteriori probability, that is, the probability that a patient will suffer from a disease after doing the test, depending on whether the result is positive or negative.

**Fig. 6:**
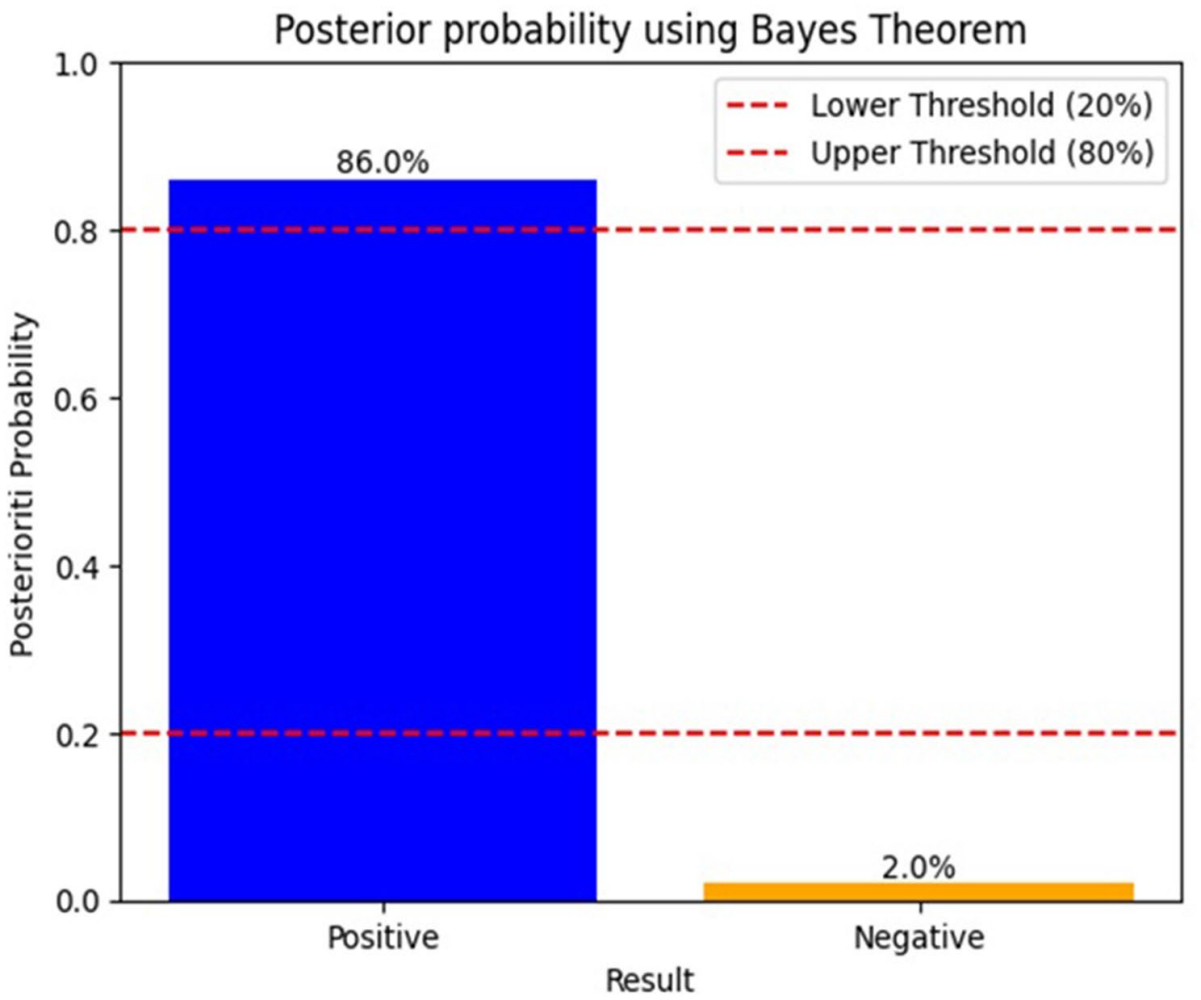
Bayesian analysis

In this test, two critical thresholds are established for decision making, a lower limit of 20% and an upper limit of 80%. When the test is positive and the prevalence is greater than 80%, it is considered that the patient should be treated because it indicates a high probability of the presence of the disease and justifies medical intervention. Figure 6.

In this study, in accordance with the results of sensitivity and specificity obtained in the study, it was found that when the test is positive, the posterior probability increases to 86%, being above the treatment line, and when it is negative, it decreases to 2%. These results provide the clinical certainty necessary for the use of the model.

**Fig. 7:**
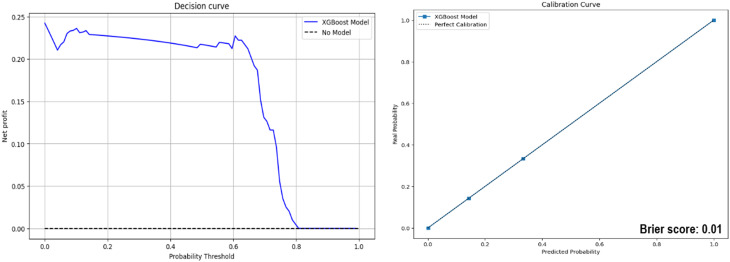
Calibration and decision curve

**Calibration and Decision curve.** The decision curve highlights the net benefit of using the XGBoost model at various probability thresholds, showing high net benefits at lower thresholds. The calibration curve demonstrates that the model’s predicted probabilities are closely aligned with the actual outcomes, supported by a low Brier score of 0.01, indicating excellent predictive accuracy (Fig. 7).

After constructing and evaluating the model, we proceeded to implement a web application to facilitate its use in a clinical setting. The application provides an easy-to-use interface for entering patient data and obtaining predictions from the XGBoost model, thus allowing physicians and other healthcare professionals to use the model in their daily clinical practice. The application can return the calculated risk of IH once all input variables are given. The application is available on the following web page: https://hi-eelh-app-1035249958862.us-central1.run.app/

## Discussion

The study’s main objective was to determine if machine learning could help develop a predictive scale for incisional hernia in postoperative patients with midline laparotomy. In this study, a predictive model was created using the XGBoost technique with better diagnostic performance than the traditional multiple logistic regression and decision tree models, compared with the metrics and Friedman ranking.

The most recent meta-analysis that evaluates the predictive power of seven scales published by Tansawet et al. [25] found that surgical site infection is the risk factor most closely related to IH with an OR of 5.54. In this cohort, this association was corroborated, and it was determined that surgical site infection increases the risk of IH with an OR of 5.3 (95% CI 3.4–8.12). Hence, future studies should consider this association since it is well-known that surgical site infection is a severe problem in abdominal surgery.

Of the seven scales reported in this meta-analysis, only two consider this association, and they do so retrospectively. It reduces its ability to predict since surgical site infection occurs after operating on the patient. One of the strengths of the proposed model is that an already validated scale was used that indicates the risk of presenting surgical site infection with good statistical performance [34], so this model can be used prospectively with a high degree of certainty since it was developed with this probability of risk the model.

A strong association between BMI and HI has also been reported. In the study cohort, patients with HI had an average BMI of 27.5 vs. 25.7 in those who did not develop it, with a statistically significant *p*-value (*p* = 0.00001), this situation meant that when choosing the best model we questioned the way in which the model was developed with logistic regression in which BMI had a negative factor in the presence of IH. Another risk factor reported in this meta-analysis in the study with the most important number of patients found that emergency surgery was the leading risk factor for this complication, with an OR of 4.65 [25].

All this coincides with the predictive variables that we included in our study and that the features importance analysis showed a more significant influence on its development, so our model is consistent with what is reported in the literature. It is also important to point out that the results of the meta-analysis mentioned above highlight a moderate predictive power of these scales due to the heterogeneity of both the sample and the variables chosen for its development.

The diagnostic performance of a test is measured in terms of sensitivity, specificity, and positive and negative predictive value; however, in general terms, the use of both positive and negative predictive values is preferred because this indicates the probability of developing a hernia if the test is positive or negative respectively. In this sense, the XGBoost model was superior, with a PPV of 0.88 (precision value) and a sensitivity of 0.90 (recall value). Thus, the XGBoost model performs better than the other models developed and compared with this cohort in the area under the curve (0.93) and predictive values, making it a helpful tool for predicting this complication.

As mentioned, Bayesian analysis based on the model results helps calculate the probability that a patient has a disease given a positive or negative diagnostic test. The Bayesian analysis of the model presented good results, exceeding the uncertainty zone in both limits, which indicates that the model has clinical impact. The decision curve corroborates these findings by showing that the use of the model is better when compared to not using any model.

One of the strengths of our study is the standardization of the sample since three significant covariates were controlled: 1. Closing with the Jenkins rule, which was performed in all patients, 2. Two-year follow-up, which is essential to determine the presence of an incisional hernia, and 3. that in all patients in whom there was doubt about the presence of an incisional hernia, either due to the size of the hernia or the complexion of the patient, tomography was performed, hence in all patients are confident of the presence of this complication, this is important because our models were confronted with this gold standard and it gives strength to the results.

Additionally, although oncological disease is a recognized risk factor for incisional hernia and was included as a predictor variable in our model, the slightly lower (non-significant) incidence of IH observed in oncological patients in this cohort (68.3% with IH vs. 75.2% without IH, *p* = 0.07) may reflect optimized perioperative management and nutritional support in many elective oncological cases. Similarly, the higher rate of stoma creation in patients undergoing colon surgery (75% vs. 24% in non-colon surgery cases) aligns with institutional practice, which frequently includes protective ileostomies (in addition to colostomies), and does not represent selection bias.

Another strength is the development of a web app that will allow the surgeons in charge of abdominal wall closure to determine the patient’s risk in real-time quickly and that the model is not based on laboratories or tests that are difficult to carry out, thus this model it can be applied in low-resource hospitals, which increases its external validity.

Some scales do not consider surgical site infection as a predictor variable because it occurs after surgery and is difficult to predict. Still, the relationship between infection and hernia is such that it should not be ignored, and perhaps this makes its power predictive less. Other scales that consider infection as a predictor evaluate it dichotomously, whether it occurred or not, because they are retrospective, and this status is already known in the cohort. Our study calculated the risk of presenting surgical site infection with the American Association of Surgeons web app, as any surgeon would do daily and before surgery. Finally, the model demonstrated significant clinical utility. Not only was the model predictive and reliable but it was also found to be practical and beneficial in a clinical setting. By accurately identifying high-risk patients, the model can aid clinicians in focusing their resources and interventions on those who most need it.

This performance in calibration, discriminative ability, and clinical utility positions our XGBoost model as a good tool for outcome prediction in our target population. Further prospective studies are needed to confirm these findings and to assess the impact of using this model in routine clinical practice.

Van Royen et al. [54] analyzes the impact of prognostic models developed with machine learning, reporting that of the published studies a small percentage ends up being used in daily medical practice, this is because there is not enough complete information to be applied or validated, their development is in a different population than what happens daily in clinical practice, they are expensive, their validation is poor or they have no impact or a negative impact on patient outcomes in impact studies. Our model has the advantage of clearly defining the measured outcomes, the variables were chosen well, and they are not costly, nor do they need to be invasive or expensive procedures and when creating the web app it is available so that surgeons can use it in their daily clinical practice. At this time in abdominal wall closure, few surgeons have adopted prophylactic measures, especially using mesh. Possible reasons for the lack of adoption are mistrust of the evidence to concern about complications, cost, and other factors [55], the present study with the model and the developed web application will provide more objective information for decision-making, and thus, the surgeon in charge of wall closure could carry out a more selective assignment of prevention interventions.

Although internal validation suggests good generalization, the main limitations of this study are its single-center, retrospective design and the need for external validation to confirm our findings in diverse populations. Currently, prospective external validation is currently underway to address generalization. In the future, secure collaborations enabled by the web application could allow anonymized real-world data collection from users, facilitating periodic recalibration of the model to mitigate potential institutional biases and further enhance performance.

## Conclusions

A predictive model was created using the XGBoost technique with better diagnostic performance than the traditional multiple logistic regression and decision tree models, compared with the metrics and Friedman ranking. The model proved highly effective, showing good capacity in discriminating and predicting IH.

The robustness of the model is related to the correct selection of the variables: the analysis of the importance of the characteristics showed that variables such as infection risk, BMI, and clinical factors such as emergency surgery have a significant impact on the model predictions. The clinical utility of the model was demonstrated with Bayesian analysis of the model results. An easy-to-use web application was developed that does not require sophisticated or expensive clinical studies.

## Electronic supplementary material.

Below is the link to the electronic supplementary material.

Supplementary material 1

Supplementary material 2

Supplementary material 3

## Data Availability

The datasets used in this study are publicly available in the Mendeley Data repository (DOI: 10.17632/s5yws2pjkn0.1).

## Abbreviations

IH: Incisional hernia
ML: Machine learning
LR: Logistic Regression
DT: Decision Tree
XGBoost: Extreme Gradient Boosting
BMI: Body Mass Index
AUC: Area under the receiver operating characteristic curve
OR: Odds ratio
CI: Confidence interval
