# Supplementary material for: Incisional hernia prediction using machine learning models
Source: BMC Med Inform Decis Mak. 2026 Feb 27;26:104. doi: 10.1186/s12911-026-03382-8 (PMC13049861; doi:10.1186/s12911-026-03382-8)
Supplement: Supplementary file 1 — Supplementary material 1 [file 12911_2026_3382_MOESM1_ESM.docx]

**Appendix A.1. Variable selection for model training for incisional hernia predictor**

Unlike published studies, where a database is filled out and analyzed to obtain the variables of the models and measure their discrimination capacity, in this study, it was decided first to choose the variables that would form the model according to the results of the eight chosen studies. Then, the database was filled out for analysis. Thus, 245 variables were obtained from eight published scales from 2000 to the present to predict incisional hernia. The model's selection of variables was based on three phases by integrating the prior knowledge from these scales: **1st Phase**: The variables did not meet the inclusion criteria. **2nd Phase**: Repeated variables, synonymous, redundant, or included in another variable. **3rd Phase:** global variables, those that presented a statistically significant difference in 2 or more scales were chosen

**Appendix A.1. Variable selection for model training for incisional hernia predictor**

| **Study** | **Sample size** | **(Total) Variables included in each study** | **1st Phase** | **2^nd^ Phase.** |
| --- | --- | --- | --- | --- |
| Fisher et al., 2016 [21] | 12373 | (38)Sex, Race, Age, Hypertension, *Obese*, BMI, Smoker, Anemia, Hyperlipidemia, Benign gynecologic mass, Cardiovascular disease, *Pulmonary disease, GI malignancy*, Renal disease, *Liver disease*, Diabetes, Intestinal obstruction, *Malnutrition, History of chemotherapy, History of radiation*, Alcohol abuse, Irritable bowel syndrome, *History GI surgery,* Systemic infection, *Acute GI inflammation, Gastric surgery, Hepatectomy, Pancreatectomy, Splenectomy, Large bowel surgery, Small bowel resection, Hysterectomy, Fistulectomy, Ostomy creation, Ostomy reversal*, History of wound complication, *Surgical site infection*, costs. | 3 | 21 |
| Basta et al., 2019 [24] | 29739 | (28)*Sex*, Race, *Age*, *BMI, Smoker, Cardiovascular disease, Pulmonary disease, Hypertension, Diabetes, Recent weight loss, Cancer, History of chemotherapy/radiation, History of drug/alcohol abuse*, Chronic anticoagulation, 2 or more Elixhauser comorbidities, Open approach, Laparoscopic approach, Open hysterectomy, Laparoscopic hysterectomy*, Emergent laparotomy, Emergent vascular surgery, Preoperative small bowel obstruction, History of abdominal surgery*, COPD, *Chronic liver disease*, ASA/anticoagulant use, *surgical site infection*, costs. | 8 | 18 |
| Cherla et al., 2017 [19] | 247 | (18) *Age, Sex*, Ethnicity, *BMI*, ASA, *Tobacco use, COPD, Diabetes mellitus, Earlier abdominal operation, Albumin, Hemoglobin, Cancer stage, Chemotherapy,* Preoperative radiographic ventral hernia, Laparoscopic or open, *Emergency surgery, Intraoperative Blood transfusion, Surgical Site Infection*. | 3 | 14 |
| Goodenough et al., 2015 [18] Incision length, | 625 | (29) *Age, Sex*, Ethnicity, Functional status, *ASA score, BMI,* Collagen vascular disorder, *Diabetes mellitus, COPD, Hypertension, Cardiovascular disease, Liver disease*, Immunosuppression*, Active smoking, Alcohol use disorder,* Hematocrit*, Albumin, Previous operation*, History of SSI, Open abdomen, Creatinine, Operative duration, Transfusion, Operative approach, Fascial incision type, Wound class, type of Suture, Closure technique, surgical site infection. | 10 | 15 |
| Basta et al., 2016 [20] | 2161 | (20) *Age*, index surgery, Race, *Gender, BMI, Diabetes, Hyperlipidemia, Hypertension, CV disease, Liver disease, Pulmonary disease, Malnutrition, Smoker, History of chemotherapy, Anemia*, Laparoscopic approach, Open approach, *History of abdominal surgery,* History of wound complication, *Concurrent GI Operation.* | 5 | 15 |
| Veljkovick et al., 2009 [17] | 603 | (43) *Age, Gender*, BMI, Diabetes, *COPD, Current tobacco use, Earlier abdominal operations,* Sagittal abdominal diameter, Transverse abdominal diameter, *History of earlier malignancy, Malignancy during or after operation, Preoperative jaundice, Preoperative anemia, Preoperative uremia*, Preoperative hypoproteinemia, Preoperative antibiotic prophylaxis, Preoperative heparin prophylaxis, *Urgent operation,* Operation conducted during work shift, Duration of operation, *Perioperative blood product transfusion, Intraabdominal infection during operation,* Mechanical ventilation, Abdominal incision length, Length of fascial suture, Ratio of suture length to incision length, Type of fascial suture, Type of laparotomy incision closure, Incisional drain, Retention sutures, Follow-up time, Incisional hernia at midline laparotomy site, Reoperation during hospitalization, *Surgical site infection*, Postoperative nausea score, Postoperative emesis score, Postoperative cough score, Postoperative pain score, Worst pain score, Duration of postoperative IV analgesic use, Duration of postoperative antibiotic use, Time to suture removal/complete epithelialization. | 21 | 13 |
| Lanni et al., 2017 [22] | 30741 | (43) Race, *Age*, Hospital type, index surgery, Length of stay, Median household income, Laparoscopic colectomy, Open colectomy, Total costs of index surgery, *Smoking, Coronary disease, Peripheral artery disease,* Hyperlipidemia, *Chronic pulmonary disease, Radiation, Malnutrition, Obesity, Congestive heart failure, Cardiac arrhythmias, Valvular disease, Pulmonary circulation disorder, Peripheral vascular disorder, Hypertension uncomplicated, Hypertension complicated*, Other neurologic disorder, *Diabetes uncomplicated, Diabetes complicated*, Hypothyroidism, *Renal failure, Liver disease, Peptic ulcer disease excluding bleeding, Metastatic cancer, Solid tumor without metastasis, Collagen vascular disease, Coagulopathy, Weight loss, Fluid and electrolyte disorder, Blood loss, anemia, Deficiency iron, Alcohol abuse*, Drug abuse, *COPD*, Benign only disease. | 8 | 31 |
| Tecce et al., 2017 [23] | 2145 | (36) *BMI, Age*, Race, *Diabetes, Hypertension, History of Smoking, Hyperlipidemia, Malnutrition, Anemia, Cardiovascular Disease, Liver Disease, Renal Disease, Pulmonary Disease, History of Chemotherapy, History of Radiation,* Operative Approach, *History of Abdominal Surgery,* History of Wound Complication, *Acute Inflammatory Process, GI Malignancy, Gynecologic + GI Surgery, Benign Pelvic Disease, Endometrial Hyperplasia, Pelvic Prolapse, Pelvic Inflammatory Disease, Gynecologic Malignancy, Malignancy of GU Tract*, Vertical Incision, *Ascites, Total or Radical Hysterectomy, Surgical Wound Complication*, Reoperation, Readmissions, Costs of Care, Follow Up (Months). | 8 | 28 |
| Total, of patient: 78634 | | Total, of variables: 245 | Removed:66 | Adjusted:145 |

**1st Phase**: variables that did not meet the inclusion criteria (66 eliminated), leaving 179 variables. These variables are underlined.

**2nd Phase**: Repeated, synonymous, or redundant or overlapping variables were merged or adjusted (145 variables adjusted), resulting in 34 final global variables. These are marked in *italic* font.

1. Sex (gender), 2. Age, 3. Hypertension (uncomplicated, complicated), 4. BMI (Obese, malnutrition, Recent weight loss, weight loss), 5. Smoker (Tobacco use, active smoking, current tobacco use), 6. Anemia (Hemoglobin, Intraoperative blood transfusion, Hematocrit, preoperative blood transfusion, preoperative anemia, deficiency iron, blood loss), 7. Hyperlipidemia, 8. Oncologic disease ( yes: malignancy after operation, metastatic, solid tumor without metastatic, GI malignance, History of chemotherapy, history of radiation, cancer, cancer stage, radiation, chemotherapy, history of earlier malignancy, Malignancy GU tract, gynecologic malignancy. No: Benign gynecology mass, Benign Pelvic Disease, Endometrial Hyperplasia, Pelvic Prolapse, Pelvic Inflammatory Disease) 9. Renal disease (creatinine, preoperative uremia, renal failure), 10. Jaundice (liver disease, chronic liver disease, preoperative jaundice), 11. Diabetes (diabetes complicated, uncomplicated), 12. Colon surgery (yes: large bowel surgery, no: intestinal obstruction, Total or Radical Hysterectomy, Peptic ulcer disease excluding bleeding, Gastric surgery, Hepatectomy, Pancreatectomy, Splenectomy, Small bowel resection, Hysterectomy, Fistulectomy), 13. Alcohol abuse (history of drug and alcohol abuse, alcohol use disorder, alcohol disorder, alcohol abuse), 14. Previous surgery (History GI surgery, history of abdominal surgery, early abdominal surgery, previous operation), 15. Emergency surgery (acute GI inflammation, emergent laparotomy, urgent surgery, emergent vascular surgery), 16. Ostomy (creation, reversal), 17. Wound infection (surgical site infection, surgical site complication, systemic infection, intraabdominal infection during operation), 18. Chronic anticoagulation, coagulopathy), 18. COPD (pulmonary disease, pulmonary chronic disorder), 19. ASA, 20. Functional status, 21. Collagen vascular disease, 22. Cardiovascular disease (cv disease, Coronary, valvular, Peripheral arterial, Peripheral vascular, congestive, Arrithmia), 23. Immunosupression, 24. Ascites (albumin, preoperative hypoproteinemia), 25. Fluid and electrolytic Disorder, 26. Hypothyroidism, 27. Po nausea score, 28. Po caught score, 29. Po emesis score, 30. Po pain score, 32. Neurological disorder, 33. Time for surgical remove suture, 34. Neurological disorder

**3rd Phase:** Of these 34 global variables, those that presented a statistically significant difference in 2 or more scales were chosen: 13 were chosen and 21 were eliminated.

Sex, age, BMI, COPD, Anemia, Ascites, Oncologic disease, Ostomy, Wound infection, Colon surgery, Previous surgery, Emergency surgery and, jaundice.
