# Supplementary material for: Incisional hernia prediction using machine learning models
Source: BMC Med Inform Decis Mak. 2026 Feb 27;26:104. doi: 10.1186/s12911-026-03382-8 (PMC13049861; doi:10.1186/s12911-026-03382-8)
Supplement: Supplementary file 2 — Supplementary material 2 [file 12911_2026_3382_MOESM2_ESM.docx]

**Appendix A.2 Diagnostic performance of ACS calculator score**

The difference in the value of the Risk wound infection calculated with the ACS calculator was analyzed. The following figures analyze the difference in means with the Bayesian analysis between both groups with and without infection, determining whether there is a correlation between the presence or not of infection and the highest score on the scale Figure 2 A. Then the diagnostic performance was evaluated with the roc curve and the maximum point of sensitivity and specificity Figure 2B.

**
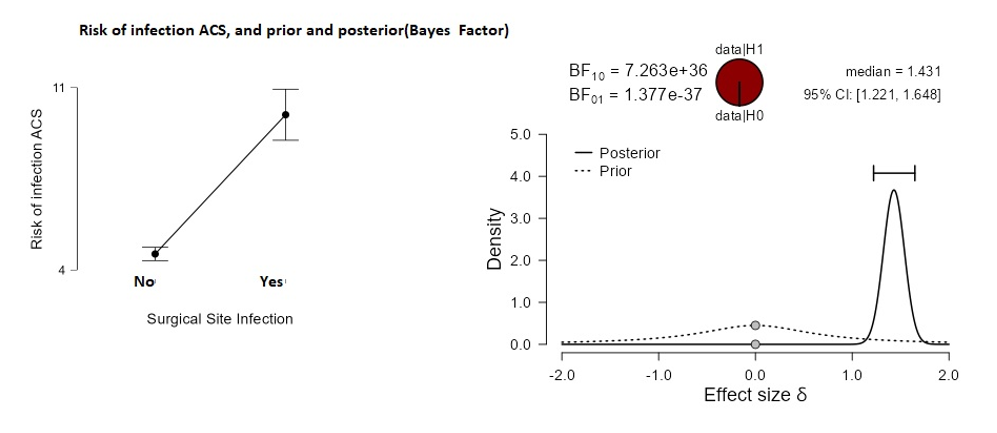
**

**Figure 2 A.** Difference of means and Bayesian analysis, ACS calculator scale score

**
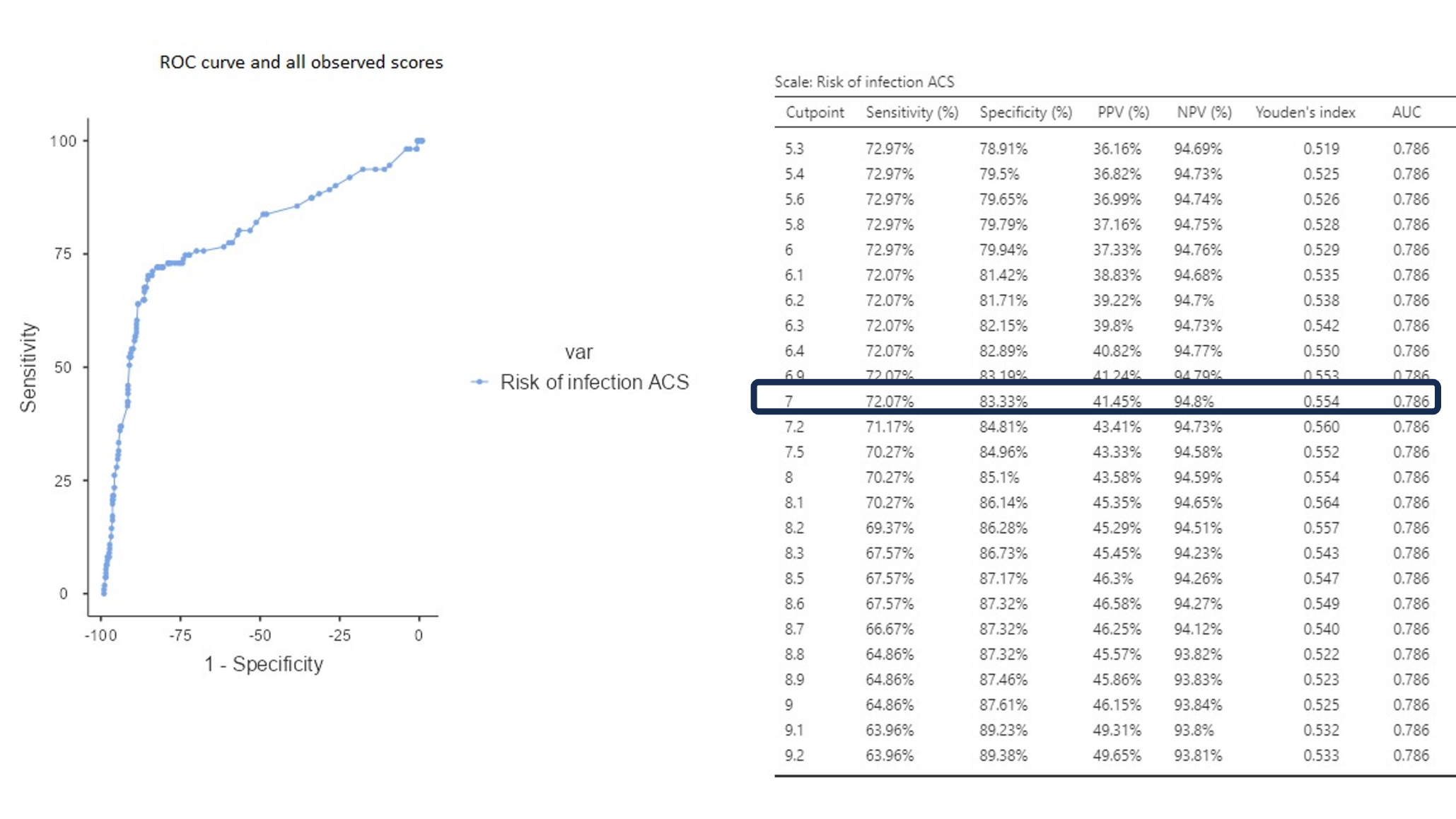
**

**Figure 2B.** ROC curve and diagnostic performance ACS calculator
