# Supplementary material for: Incisional hernia prediction using machine learning models
Source: BMC Med Inform Decis Mak. 2026 Feb 27;26:104. doi: 10.1186/s12911-026-03382-8 (PMC13049861; doi:10.1186/s12911-026-03382-8)
Supplement: Supplementary file 3 — Supplementary material 3 [file 12911_2026_3382_MOESM3_ESM.docx]

**Appendix A.3** Sample sizes calculated.

- Using the pmsampsize library in the R studio programming environment with a binary-type result
- looking for a c-statistic value of 0.9, with 13 predictive parameters and a prevalence of 0.21 in the presence of IH, assuming a Cox-Snell R-sq = 0.33
- a 0.05 difference between the apparent and fitted R-squared, and a 0.05 difference in margin of error in the intercept estimate

| Samp size Shrinkage Parameter CS Rsq Max Rsq Nag Rsq EPP |
| --- |
| Criterio 1 296 0.900 13 0.3395 0.642 0.529 4.44 |
| Criterio 2 349 0.914 13 0.3395 0.642 0.529 5.23 |
| Criterio 3 255 0.914 13 0.3395 0.642 0.529 3.82 |
| Final 349 0.914 13 0.3395 0.642 0.529 5.23 |

Resulting in a sample size of 349 patients for the development of the model based on obtaining 74 events (assuming a prevalence of 21%) and an event ratio of 5.23 for each parameter used in the development of the model.
